# The Biological Big Bang model for the major transitions in evolution

**DOI:** 10.1186/1745-6150-2-21

**Published:** 2007-08-20

**Authors:** Eugene V Koonin

**Affiliations:** 1 National Center for Biotechnology Information, National Library of Medicine, National Institutes of Health, Bethesda, MD 20894, USA

## Abstract

**Background:**

Major transitions in biological evolution show the same pattern of sudden emergence of diverse forms at a new level of complexity. The relationships between major groups within an emergent new class of biological entities are hard to decipher and do not seem to fit the tree pattern that, following Darwin's original proposal, remains the dominant description of biological evolution. The cases in point include the origin of complex RNA molecules and protein folds; major groups of viruses; archaea and bacteria, and the principal lineages within each of these prokaryotic domains; eukaryotic supergroups; and animal phyla. In each of these pivotal nexuses in life's history, the principal "types" seem to appear rapidly and fully equipped with the signature features of the respective new level of biological organization. No intermediate "grades" or intermediate forms between different types are detectable. Usually, this pattern is attributed to cladogenesis compressed in time, combined with the inevitable erosion of the phylogenetic signal.

**Hypothesis:**

I propose that most or all major evolutionary transitions that show the "explosive" pattern of emergence of new types of biological entities correspond to a boundary between two qualitatively distinct evolutionary phases. The first, inflationary phase is characterized by extremely rapid evolution driven by various processes of genetic information exchange, such as horizontal gene transfer, recombination, fusion, fission, and spread of mobile elements. These processes give rise to a vast diversity of forms from which the main classes of entities at the new level of complexity emerge independently, through a sampling process. In the second phase, evolution dramatically slows down, the respective process of genetic information exchange tapers off, and multiple lineages of the new type of entities emerge, each of them evolving in a tree-like fashion from that point on. This biphasic model of evolution incorporates the previously developed concepts of the emergence of protein folds by recombination of small structural units and origin of viruses and cells from a pre-cellular compartmentalized pool of recombining genetic elements. The model is extended to encompass other major transitions. It is proposed that bacterial and archaeal phyla emerged independently from two distinct populations of primordial cells that, originally, possessed leaky membranes, which made the cells prone to rampant gene exchange; and that the eukaryotic supergroups emerged through distinct, secondary endosymbiotic events (as opposed to the primary, mitochondrial endosymbiosis). This biphasic model of evolution is substantially analogous to the scenario of the origin of universes in the eternal inflation version of modern cosmology. Under this model, universes like ours emerge in the infinite multiverse when the eternal process of exponential expansion, known as inflation, ceases in a particular region as a result of false vacuum decay, a first order phase transition process. The result is the nucleation of a new universe, which is traditionally denoted Big Bang, although this scenario is radically different from the Big Bang of the traditional model of an expanding universe. Hence I denote the phase transitions at the end of each inflationary epoch in the history of life Biological Big Bangs (BBB).

**Conclusion:**

A Biological Big Bang (BBB) model is proposed for the major transitions in life's evolution. According to this model, each transition is a BBB such that new classes of biological entities emerge at the end of a rapid phase of evolution (inflation) that is characterized by extensive exchange of genetic information which takes distinct forms for different BBBs. The major types of new forms emerge independently, via a sampling process, from the pool of recombining entities of the preceding generation. This process is envisaged as being qualitatively different from tree-pattern cladogenesis.

**Reviewers:**

This article was reviewed by William Martin, Sergei Maslov, and Leonid Mirny.

## Open peer review

This article was reviewed by William Martin, Sergei Maslov, and Leonid Mirny.

## Background

### The enigmatic nexuses

The famous single illustration of Darwin's "Origin of Species" shows generalized binary trees. According to Darwin, "The affinities of all the beings of the same class have sometimes been represented by a great tree. I believe this simile largely speaks the truth." [1] Darwin's notion of a tree as a valid depiction of evolution became the foundation of the grand metaphor of the tree of life (TOL) that had been propounded as a generally adequate depiction of the entire history of life, above all, by Haeckel who expanded Darwin's schematic into an arborescent and picturesque tree [2].

However, the evolution of life is, obviously, a non-uniform process as described, e.g., in Simpson's classic book [3,4], and captured, more formally, in the punctuated equilibrium concept of Gould and Eldredge [5,6]. Lengthy intervals of gradualist modification are punctuated by brief bursts of innovation that are often called transitions, to emphasize the fact that they culminate in the emergence of new levels of organizational and functional complexity [7]. Although it is hardly feasible to compile a definitive list of biological transitions, certain events, such as the origin of the first cells, the origin of the eukaryotic cell, or the origin of multicellular plants and animals, definitely qualify as major transitions. The term "transition" might not be the most precise descriptor of the pivotal events in life's evolution because the emerging new forms do not necessarily (or, even, typically) replace pre-existing ones (e.g., eukaryotes do not replace prokaryotes). Instead, in these evolutionary (or, perhaps, more precisely, revolutionary) nexuses, forms with a new level of organizational complexity emerge that, subsequently, coexist with the older, simpler forms. Nevertheless, with this understanding, I use the commonly accepted term "transitions" through the rest of this article.

**Figure 1:**
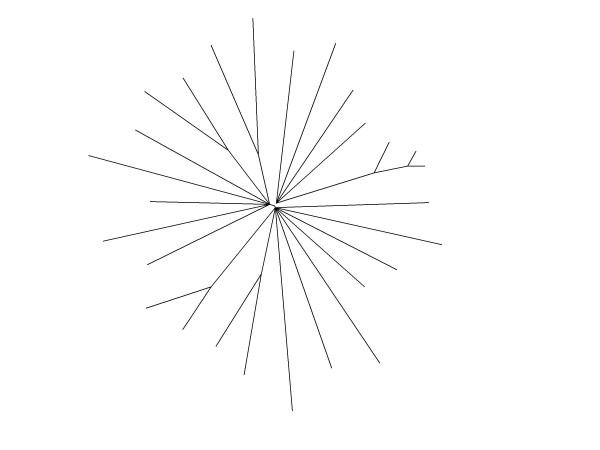
A Bush of Life: a typical tree with unresolved deep branches. The tree was generated from simulated data using the TreeView program [114].

There seems to be a striking commonality between all major transitions in the evolution of life. In each new class of biological objects, the principal types emerge abruptly, and intermediate grades (e.g., intermediates between the precellular stage of evolution and prokaryotic cells or between prokaryotic and eukaryotic cells), typically, cannot be identified. The events that lead to the emergence of a new level of complexity and, obviously, are crucial in the evolution of life elude representation through a unique tree topology and are notoriously hard to reconstruct. Whatever trees have been constructed for these stages of life's history, have extremely short, most often, unreliable internal branches, and the tree topology tends to differ for different genes [8] (Fig. 1). Below I list the most conspicuous instances of this pattern of discontinuity in the biological and pre-biological domains, and outline the central aspects of the respective evolutionary transitions.

#### 1. Origin of protein folds

There seem to exist ~1,000 or, by other estimates, a few thousand distinct structural folds the relationships between which (if existent) are unclear [9-11].

#### 2. Origin of viruses

For several major classes of viruses, notably, positive-strand RNA viruses [12] and nucleo-cytoplasmic large DNA viruses (NCLDV) of eukaryotes [13,14], substantial evidence of monophyletic origin has been obtained. However, there is no evidence of a common ancestry for all viruses [15].

#### 3. Origin of cells

The two principal cell types (the two prokaryotic domains of life), archaea and bacteria, have chemically distinct membranes, largely, non-homologous enzymes of membrane biogenesis[16,17], and also, non-homologous core DNA replication enzymes [18]. This severely complicates the reconstruction of a cellular ancestor of archaea and bacteria and suggests alternative solutions [16,19].

#### 4. Origin of the major branches (phyla) of bacteria and archaea

Although both bacteria and archaea show a much greater degree of molecular coherence within a domain than is seen between the domains (in particular, the membranes and the replication machineries are homologous throughout each domain), the topology of the deep branches in the archaeal and, especially, bacterial phylogenetic trees remains elusive. The trees conspicuously lack robustness with respect to the gene(s) analyzed and methods employed, and despite the considerable effort to delineate higher taxa of bacteria [20-23], a consensus is not even on the horizon. The division of the archaea into two branches, euryarchaeota and crenarchaeota is better established but even this split is not necessarily reproduced in trees, and further divisions in the archaeal domain remain murky [23-25].

#### 5. Origin of the major branches (supergroups) of eukaryotes

Despite many ingenious attempts to decipher the branching order near the root of the phylogenetic tree of eukaryotes, there has been little progress, and an objective depiction of the state of affairs seems to be a "star" phylogeny, with the 5 or 6 supergroups established with reasonable confidence but the relationship between them remaining unresolved [26-31].

#### 6. Origin of the animal phyla

The Cambrian explosion in animal evolution during which all the diverse body plans appear to have emerged almost in a geological instant is a highly publicized enigma [32-35]. Although molecular clock analysis has been invoked to propose that the Cambrian explosion is an artifact of the fossil record whereas the actual divergence occurred much earlier [36,37], the reliability of these estimates appears to be questionable [38]. In an already familiar pattern, the relationship between the animal phyla remains controversial and elusive.

The bushes in the tree of life (TOL) recently have been examined in some detail, and their appearance has been attributed, primarily, to cladogenesis compressed in time that appears to be characteristic of transitional epochs in evolution. Also, the erosion of the phylogenetic signal inevitably results in poor resolution of phylogenetic trees for ancient divergence events like all those listed above. Nevertheless, it is generally assumed that, in principle, the TOL exists and is resolvable although, in practice, full resolution might never be attained and, furthermore, might not even be particularly important for understanding the actual events that transpired during the respective transitional stages [8,39].

Here, I argue for a fundamentally different solution, i.e., that a single, uninterrupted TOL ***does not exist*, **although the evolution of large divisions of life for extended time intervals can be adequately described by trees. I suggest that evolutionary transitions follow a general principle that is distinct from the regular cladogenesis. I denote this principle the Biological Big Bang (BBB) Model. Under this model, each of the biological transitions is, indeed, a transition in a more specific, technical sense, i.e., a switch between two phases of evolution, a phase of rapid evolution (inflation) characterized by rampant exchange and recombination of genetic material, followed by congealing into a relatively slow phase governed by the tree pattern. This principle shows substantial analogies with the new model of universe nucleation (a radical re-interpretation of the classic Big Bang) that is part of the eternal inflation cosmology.

### Unconventional solutions

In a seminal 1998 paper, Carl Woese proposed that the early stages of life's evolution including that of the Last Universal Cellular Ancestor (LUCA), involved rampant horizontal exchange of genetic material between primordial life forms such that individual lineages could not form [40,41]. As aphoristically formulated by Woese, "This communal ancestor has a physical history but not a genealogical one" [40]. The lineages were thought to emerge as the "temperature" (used here as a physical metaphor describing the changing intensity of the genetic exchange) of the evolving mixture lowered and the functional systems of cells "crystallized" (again, metaphorically) one by one.

Subsequently, the notion of a "communal ancestor" has been developed into a specific scenario [16,19] that derived from the comparative-genomic indications that the enzymes of membrane biogenesis and the core DNA replication machineries in archaea and bacteria were non-homologous [18,42]. Under this scenario, LUCA was a diverse population of genetic entities (initially, RNA, subsequently, a mixture of RNA and DNA segments) that inhabited networks of inorganic compartments at hydrothermal vents. Extensive exchange of genetic content between compartments, the primordial analog of HGT, is an inherent feature of this model. A transition from selection for individual genetic entities to the selection for "selfish cooperatives" (in particular, compartment contents) is thought to have occurred in this system, followed by independent escape of the first membrane-bounded cells [19]. There might have been numerous "attempted" escapes but only two ultimately successful ones, leading to archaea and bacteria.

The scenario of evolution of non-membrane-bounded but compartmentalized populations of diverse genetic elements has been extended and elaborated to include viruses, and developed into the virus world concept [15]. The notion of the virus world stems, primarily, from the fact that a set of genes encoding essential proteins involved in viral genome replication, packaging, and virion formation (virus hallmark genes [15]), are shared by numerous groups of dissimilar and, otherwise, apparently, unrelated viruses. The early, "communal" stage of evolution is envisaged as, essentially, virus-like where, initially, the progenitors of viral and cellular genomes were indistinguishable but, gradually, the segregation of bona fide parasites (the would be viruses) and selfish cooperatives (the would be cellular life forms) took hold. The major classes of extant prokaryotic viruses are thought to have emerged directly from the primordial pool of genetic elements. In addition, it has been proposed that eukaryogenesis was "the second melting pot" of virus evolution where the major groups of eukaryotic viruses emerged, primarily, through recombination between various bacteriophage and cellular genomes.

In a striking parallel, Lupas et al. have proposed that modern protein folds evolved by recombination of ancient peptide modules, such that the folds have independent and polyphyletic origins although they all ultimately derive from the same recombining pool of genetic elements encoding primordial peptides [43]. The recent demonstration (in simulation studies) that high concentration of RNA would have been readily attainable in networks of inorganic compartments at hydrothermal vents [44] has added credibility to the notion that that recombination and fusion of RNA molecules could have played a major role even in pre-biological evolution [45,46].

The realization that HGT is extremely widespread among prokaryotes [47-52], which was one of the principal early discoveries of the genomic revolution [53], led to a reappraisal of the TOL concept [54-57]. As noticed by Ford Doolittle, as long as the HGT is quantitatively substantial and involves, to a lesser or greater extent, all categories of genes, "Molecular phylogeneticists will have failed to find the "true tree," not because their methods are inadequate or because they have chosen the wrong genes, but because the history of life cannot properly be represented as a tree." [56]. It has been argued, however, that the TOL still could be saved, even in the presence of extensive HGT, by redefining the TOL as the consensus tree of the relatively stable (refractory to HGT) core of highly conserved genes [22].

In recent general, philosophically oriented treatises, O'Malley and Boucher, and Doolittle and Bapteste [58,59] further question the validity of the TOL paradigm. Emphasizing the plurality of process in biological evolution, which includes both vertical, tree-like inheritance and exchange of genetic material between diverse life forms, traditionally viewed as HGT (although the changing paradigm of microbiology would blur the very distinction between the two processes [58]), Doolittle and Bapteste call for replacing the tree-centered monism by a plurality of pattern in evolutionary models [59]. The scenarios of early evolution outlined above fit that bill by postulating that the early, pre-cellular evolution of life followed a pattern that was qualitatively distinct from the pattern of subsequent evolution. The principal feature of the distinct, early phase of evolution is thought to be the extensive exchange of genetic material that assumes different forms, results in rapid evolutionary innovation, and precludes the formation of distinct lineages. Here, I generalize on this pattern by proposing that a phase of rapid, promiscuous evolution might underlie many, if not most of the major transitions in the history of life. I draw a fundamental analogy between these transitional stages of biological evolution and the birth of universes from Big Bang events as interpreted in the eternal inflation model of cosmology.

## Hypothesis: the Biological Big Bang model of evolutionary transitions

### The major transitions in biological evolution as Biological Big Bang events

**Figure 2:**
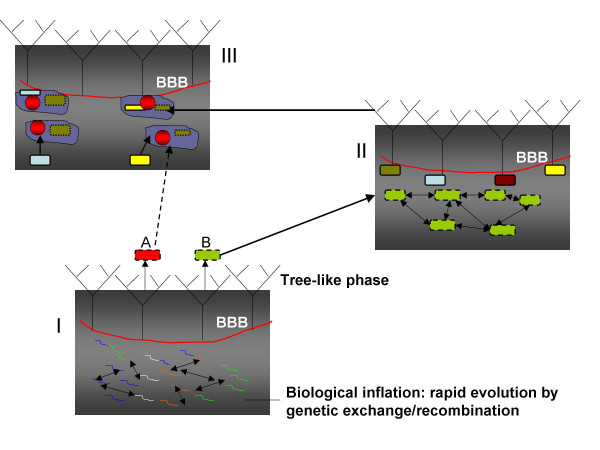
Biological Big Bangs and the emerging pattern of tree-like evolution: transitions between rapid and slow phases of evolution in the history of life. The transition between the rapid, inflationary, and slow, tree-like, phases of evolution is shown by the red line and denoted BBB. The similarity to the depiction of Big Bang events in the evolution of the multiverse in Fig. 3 is deliberate. I. The pre-cellular BBBs. The squiggles of different colors denote genetic elements in the primordial gene pool, and arrows denote recombination/fusion processes. The emerging trees are those of individual genes and virus-like agents. The emergence of the proto-archaeal (A) and proto-bacterial (B) cells is shown as well. II. Origin of the major bacterial lineages. The rounded rectangles show proto-bacterial cells with leaky membranes (broken lines). The arrows denote extensive horizontal gene transfer. The colored shapes denote emerging bacterial lineages (trees) with tighter membranes (solid lines). A similar schematic for the origin of archaeal lineages is not shown. III. Origin of the eukaryotic supergroups. The irregular shapes show proto-eukaryotic cells that already harbored mitochondria derived from α-proteobacteria (dark green shapes inside – shown to derive from one of the bacterial trees supposed to correspond to the proteobacterial lineage) and have evolved nuclei (red spheroids, so colored to emphasize the archaeal connection). The archaeal ancestry of eukaryotes is denoted by a broken arrow (the intermediate, inflationary phase is omitted). Arrows show secondary symbioses with other bacteria or primitive eukaryotes (colored shapes) that are postulated to give rise to the eukaryotic supergroups (trees).

I hypothesize that each major biological transition is, actually, a transition between two qualitatively different phases of evolution, an initial rapid phase and the subsequent, slower phase during which the tree pattern emerges (Fig. 2). The entire history of life, then, should be depicted not as a TOL but as a succession of alternating rapid and slow phases such that each new rapid phase emerges from a pre-existing tree and, in turn, gives rise to a new tree phase (Fig. 2). In modern cosmology, there is a striking analogy to this pattern, namely, the transition from the rapid, exponential expansion (inflation) of the multiverse to the much slower expansion occurring during the nucleation of an individual universe (see below). This transition corresponds to the Big Bang of the traditional model of an expanding universe. Hence I denote the transitional events in the evolution of life the Biological Big Bangs (BBBs) and refer to the rapid stages of evolution as the inflationary phase. I elaborate on this analogy in the next section.

The principal underlying force of the inflationary phases of evolution is extensive genetic exchange between the respective biological entities, taking the forms of recombination, fusion, and fission; the specifics of these processes differ for different inflationary stages. The process leading to the emergence of a new generation of increasingly complex entities at the end of each inflationary stage (i.e., the BBBs) is best described as continuous ***sampling ***of numerous combinations of genetic elements. Once, by chance, a stable, highly efficient combination emerges, a lineage of the new generation is born (Fig. 2).

Table 1 lists the putative major BBB events in the history of life on earth. The first four of the BBBs date back to the pre-cellular era:

**Table 1:** Major transitions in the history of life and proposed Biological Big Bang events

Transition/BBB	Nature of the inflationary phase (dominant genetic exchange processes)	Specifics/comments	References
Emergence of complex RNA molecules and protein folds	Recombination/fusion/fission, in the primordial gene pool, between genetic elements encoding short peptides and/or unstructured proteins, or RNA structural elements.	The first of the three (along with the origins of viruses and cells) original, great BBBs that might have shared a physical substrate, the primordial gene pool, probably, abiogenically compartmentalized. This BBB would give rise to the tree pattern of evolution (gene trees) for the first time in the history of life.	[43, 46, 115]
Emergence of the major classes of viruses	Recombination and fusion, in the primordial gene pool, of genetic elements encoding hallmark viral genes.	The second of the three great BBBs occurring in the primordial gene pool.	[15]
Emergence of the two prokaryotic cell types, archaea and bacteria	Recombination, fusion, and sorting of diverse genetic elements in the primordial gene pool.	The third and last of the three great BBBs occurring in the primordial gene pool. Crucial processes involve the formation of selfish cooperatives, extensive transfer of genetic material between compartments, and sampling of genes into emerging protocells. Probably, numerous trials on cell formation, with only two types fixed.	[15, 16, 19, 40, 41]
Emergence of the major lineages of archaea and bacteria	Extensive gene exchange between protoarchaeal and protobacterial cells with leaky membranes within primordial microbial mats, possibly, in the vicinity of hydrothermal vents.	Continued, albeit more constrained process of gene sampling, with numerous trials on more robust cells capable of departing the primordial mats.	
Emergence of the eukaryotic cell and the supergroups of eukaryotes	Extensive gene flow from endosymbionts to the host chromosome(s) accompanied by massive invasion of introns and pervasive genome rearrangement. Distinct symbiotic events giving rise to the 5 supergroups of eukaryotes.	The 5 eukaryotic supergroups are: 1. Plantae (green plants, green algae, red algae) 2. Chromalveolates (alveolates, including Apicomplexa, dinoflagellates, and ciliates, and stramenophiles including diatoms, oomycetes and many other groups) 3. Unikonts (Animals, fungi, Amoebozoa) 4. Rhizaria (Foraminifera and a variety of other, poorlycharacterized groups) 5. Excavates (kinetoplastids, euglenids, diplomonads, trichomonads, and other, poorly characterized groups) [28]. The chloroplast symbiosis, obviously, gave rise to Plantae, and a symbiosis between a primitive unicellular eukaryote and a red alga led to the emergence of the Chromalveolata. The remaining endosymbiotic events that are postulated to underlie the emergence of other supergroups might not have left morphologically distinct vestiges	[67, 70]
Origin of the major lineages within supergroups?	Invasion of mobile elements; rewiring of regulatory networks; more?		

1. the emergence of complex RNA molecules, e.g., rRNA, by fusion/recombination of smaller RNA segments;

2. the emergence of protein folds by recombination and fusion of RNA segments encoding primordial peptides and/or non-globular primordial polypeptides containing small structured units;

3. the emergence of the major classes of virus-like agents (the progenitors of the prokaryotic viruses) via recombination and fusion of primordial genetic elements;

4. the emergence of archaeal and bacterial cells, via a gene sampling process, from the same primordial pool of genetic elements at a later stage.

The existence of an initial rapid phase of evolution, characterized by extensive mixing and matching of genetic elements, has already been proposed for each of these transitions in previous studies (see above and Table 1). The recent demonstration that the primordial pool of RNA molecules might have been highly concentrated [44], creating favorable conditions for recombination and fusion [46], lends further credibility to these scenarios. Conceivably, the first trees ever to emerge during life's history were gene trees which consolidated at the first two BBBs that gave rise to complex RNA molecules and major protein folds.

The next two BBBs correspond to the emergence of the major lineages of bacteria and archaea. As indicated above, attempts to resolve the relationships between these lineages through conventional phylogenetic tree building or through analysis of rare characters (cladistics) yield conflicting and hardly compelling results. I suggest that the major lineages of bacteria and archaea are, actually, not linked by trees. Instead, the emergence of archaea and bacteria via the respective BBBs would have been followed by new inflationary phases in which the archaeal and bacterial cells formed distinct communities of cells that extensively exchanged genetic material. Conceivably, the membranes of these earliest archaea and bacteria were substantially more leaky than those of modern prokaryotes [60], making the cells highly susceptible to DNA uptake. The intensity of genetic exchange was high enough to preclude the formation of individual lineages. At this stage of evolution, archaeal and bacterial cells existed as physical entities (and communities), but conditions for the formation of cell lineages did not, and selection affected genes and gene complexes rather than cells. Evolution would be rapid, fueled by the rampant gene flow between cells, albeit not as rapid as the gene exchange between inorganic compartments during the pre-cellular inflationary epoch. In concrete, physical terms, the proto-bacteria and proto-archaea might have thrived as colonies at hydrothermal vents, outside the networks of inorganic compartments from which the first cells have "hatched" [16,19]. The proto-bacteria and proto-archaea would form physically distinct communities, in principle, similar to the present-day microbial mats[61,62], with the intensity of the gene flow between these being substantially lower than that within each of the communities. Individual bacterial and archaeal cellular lineages would emerge when gene sampling yielded selectively advantageous combinations with tighter membranes, thus, curtailing HGT. Escapes of the emerging distinct cellular forms from the communities would result in partial isolation of the lineages, and in dissemination of distinct bacteria and archaea in a variety of habitats.

The origin of the eukaryotic cell and the emergence of eukaryotic supergroups can be reasonably perceived as a single BBB event. The debate around the origin of eukaryotes continues to rage [63-65]. Nevertheless, the most parsimonious scenario has the symbiosis between an archaeon and an α-proteobacterium (the progenitor of mitochondria) as the triggering event of eukaryogenesis [65-67]. Conceivably, this event instigated a new wave of inflation during which the archaeal host DNA was bombarded by the genes and selfish elements from the symbiont, apparently, leading to dramatic rearrangements within the hybrid cell, starting with the formation of the endomembranes and the nucleus [67,68]. The existence of an extremely eventful, even if temporally brief, "stem" (i.e., preceding the divergence of the supergroups) phase of eukaryotic evolution is clearly demonstrated by the existence of numerous pan-eukaryotic sets of paralogous genes that evolved at this stage [69]. During the stem phase, the emerging eukaryotic cell, probably, evolved the phagocytic capacity and would engulf additional bacteria, typically, resulting in multiple, transient symbioses and transfer of a varying number of the bacterial symbiont's genes to the eukaryotic genome. Effectively, this is the "you are what you eat" scenario proposed by Doolittle [70], with the difference that the mitochondrial endosymbiosis is perceived as the event that triggered all aspects of eukaryogenesis, in particular, the evolution of the phagocytic capacity. A relatively stable symbiosis would result in the termination of inflation for a host subpopulation and the emergence of a new cellular lineage, one of the eukaryotic supergroups.

Currently, the evolutionary tree of eukaryotes is best represented as a bush (polytomy) of 5 or, possibly, 6 supergroups [28,31](Table 1). There is no consensus on the relationship between the supergroups. It is considered certain that the Plantae supergroup, which includes glaucophytes, red algae, green algae, and green land plants derived from the latter, emerged following the endosymbiosis of a cyanobacterium (the future chloroplast) with an ancestral eukaryotic eukaryotic cell (which, certainly, already possessed the mitochondrion) [71-74]. Furthermore, under the so-called chromalveolate hypothesis [75,76], a secondary endosymbiosis, namely, the engulfment of a red alga by a non-photosynthetic eukaryotic cell, gave rise the to Chromalveolata supergroup [74,77,78]. Several additional secondary symbioses that involve the engulfment of a photosynthetic eukaryote by a non-photosynthetic appear to have occurred at the base of several major lineages belonging to different supergroups, e.g., euglenids, diatoms, and dinoflagellates [74,77,79]. Furthermore, a remarkable variety of transient and stable bacterial symbionts have been detected in numerous individual unicellular eukaryotes [80], indicating that establishment of a symbiosis is relatively common, although evolution of symbionts into bona fide organelles appears to be much harder and rare. I propose that each of the eukaryotic supergroups emerged from the ancestral population of early eukaryotes through a distinct symbiosis with bacteria, as in Plantae, or between primitive eukaryotes themselves, as appears to be the case for Chromalveolata. Other than in Plantae and Chromalveolata, these postulated, ancient endosymbiotic events, apparently, have not left vestiges in the form of morphologically distinct organelles (at least, not widespread ones); however, traces of such events in the form of genes transferred to the nuclear genome could exist and can be sought. It should be noticed that, under the chromalveolate hypothesis, secondary loss of endosymbionts is not uncommon: apparently, it occurred independently in multiple branches of Chromalveolata, such as the ciliates and several lineages of heterkonts, dinoflagellates, and apicomplexans [72,74,77]. Thus, losses of morphologically distinguishable endosymbionts in other supergroups do not appear implausible either. The chromalveolate hypothesis remains a subject of debate, and it cannot be ruled out that it erroneously oversimplifies the history of secondary endosymbiotic events that have occurred in eukaryotic evolution [28,74,77]; this would not, however, bear upon the generality of endosymbiosis coninciding with BBB in the context of the present argument.

### The cosmological connection: eternal inflation and the Big Bang as a transition between two phases in the evolution of the multiverse

**Figure 3:**
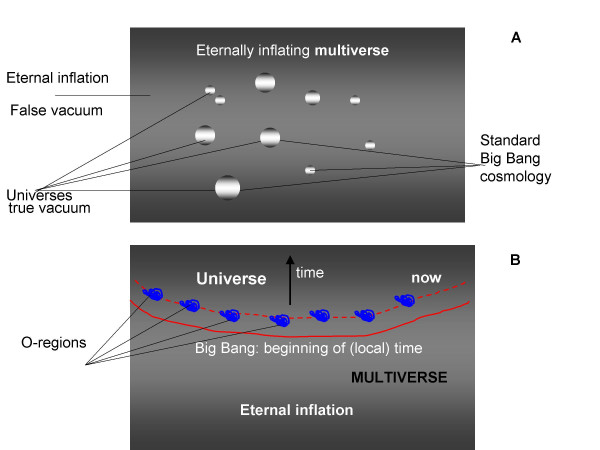
The cosmological model of eternal inflation. A. Emergence of universes as nucleating bubbles of low-energy vacuum in the eternally inflating sea of false vacuum. B. A Big Bang event at the origin of a universe: views from the inside of the emerging universe and from the outside.

In this section, I elaborate on the, apparently, deep analogies between the BBB model and the cosmological model of eternal inflation. First, however, the eternal inflation model and they way it reinterprets the Big Bang need to be briefly introduced. The dominant 20^th ^century concept of the origin and evolution of our universe, implicit in the Friedmann's solutions of general relativity equations and developed in the seminal work of Gamow and coworkers, involved expansion of the spacetime and energy-matter from an initial state characterized by values of curvature, density, and temperature that tend to infinity (mathematically, a singularity) [81,82]. The primordial "explosion" of the initial state has been dubbed (originally, sarcastically, by the astrophysicist Fred Hoyle, a staunch opponent of the very idea of an expanding universe) the Big Bang. The Big Bang model of the evolution of the universe is considered to be, effectively, proved by the discovery of the low-energy cosmic microwave background radiation (CMB) [82]. However, the nature of the Big Bang event had not received a coherent explanation before the advent, in 1981, of a new generation of cosmological models that stem from the concept of inflation. Inflation is the exponentially fast initial expansion of a universe [83-85]. Inflation is in an excellent agreement with several crucial results of observational cosmology [85,86]. In the most plausible, self-consistent inflationary models, inflation is eternal, with an infinite number of island (pocket, bubble) universes (hereinafter, simply, universes) emerging through the decay of small regions of the primordial "sea" of false vacuum and comprising the infinite multiverse [87-89](Fig. 3A). Importantly, the "populated landscape" version of string theory independently yields a very similar model of the multiverse [90-93]. Thus, although the model of eternal inflation cannot be considered proved, this is the strongly preferred current scenario of the cosmic evolution.

In the eternal inflation cosmology, the Big Bang is radically reinterpreted. Instead of being the actual beginning of time and history, a Big Bang becomes a local transition between two phases of the multiverse's evolution, namely, the rapid inflation, driven by the negative pressure of the high energy (false) vacuum, and the relatively slow expansion of an individual universe. The formation of complex structures, such as galaxies, is possible only in the second phase, but depends on quantum fluctuations of the scalar field of the decaying false vacuum at the end of inflation in the given region of the multiverse [85,86,89].

The analogy between this new picture of the Big Bang (but not the classical one) and the BBB model is apparent and straightforward. The central feature of both processes is the transition between a "hot" phase of rapid change and a "cooler" phase of slower evolution during which the formation of structures becomes possible. The emergence of a new generation of biological entities as a result of "cooling and condensation" of parts of the inflating pool of previous-generation elements is analogous to the decay of small regions of false vacuum in the inflating multiverse – the multiple Big Bang events – giving rise to individual (variously denoted as island, pocket, or bubble) universes. The decay of false vacuum can be formally described as a first order phase transition (e.g., water boiling) [94-96]. To quote the picturesque description given by Guth, "...bubbles of the new phase materialize randomly in space, just as bubbles of steam form randomly in water heated on the stove...The rate at which the bubbles materialize depends very sensitively on the details of the theory, so the decay of the false vacuum can be very fast, or very slow, or something in between." [85] This reads like a perfect coarse-grain description of a BBB; it remains to be seen whether the corresponding mathematical theory can be developed.

Of course, the analogy between the rapid phases of biological evolution and cosmological inflation and the corresponding phase transitions is not a direct, physical one but pertains to the common general characteristics of these processes. Similarly to the exponential space expansion during inflation, the brief stages of rapid evolution in the history of life are characterized by a rapid expansion of the biological informational space fueled, primarily, by various forms of recombination, fusion, and fission of genetic entities (Fig. 3). The evolutionary momentum created by these processes is analogous to the repulsive (negative) pressure of the false vacuum.

Under the eternal inflation model, all the specifics of the evolution of each universe are determined by quantum fluctuations of the scalar field of the decaying false vacuum [89]. Hence the events occurring in a particular universe cannot be predicted from any events preceding the respective Big Bang. This essential unpredictability is paralleled by the random emergence of lineages from the sea of biological inflation although, in this case, the randomness involved is, of course, deterministic rather than quantum.

There are, certainly, substantial distinctions between the BBB model and the eternal inflation cosmology. The enormous differences in scales and energies involved are obvious. Besides, under the eternal inflation model, the spacetime of the multiverse appears as an infinite sea of eternally and continuously inflating false vacuum, speckled with nucleating bubbles of universes (Fig. 3A). By contrast, in biological evolution, inflation appears to be recurring and discontinuous (Fig. 2).

To avoid confusion, it should be noted that inflationary cosmology appears to be relevant to our understanding of biological evolution at more than one level. In a recent paper, I explored direct implications of eternal inflation for the origin of replication, translation, and biological evolution itself [97]. By contrast, here, the cosmological models are used as an analogy; however, I believe that it is a powerful analogy that can substantially inform our understanding of the fundamentals of biological evolution.

## Discussion and Conclusion

The essence of the Biological Big Bang model is that evolution consists of two, fundamentally different, alternating phases which are underpinned by sharply distinct processes:

i) the rapid, inflationary phase that is, typically, characterized by extensive fusion, fission, and recombination of genetic entities

ii) the slower phase that takes over when inflation stops in a part of a community of recombining genetic elements and includes the emergent tree pattern of evolution (Fig. 2).

Herein lies the deep analogy with the eternal inflation model of the evolution of the multiverse in which universes repeatedly emerge as a result of a local halt of inflation caused by the decay of regions of false vacuum (Fig. 3A). The local termination of inflation is a phase transition (in the precise physical sense) that ushers in the second, slower phase of expansion during which, in many universes with the conducive values of the key parameters (such as the cosmological constant and the amplitude of the cosmic microwave background radiation), structures, such as clusters of galaxies, emerge [98]. This process appears analogous to the emergence of tree-like evolution in BBBs. Of course, the analogy between BBBs events and the cosmological Big Bangs leading to the birth of universes should not been construed in direct physical terms but rather pertains to the general features of the corresponding processes, in particular, the existence of a rapid and slow phases of evolution separated by a phase transition.

The major corollary of the BBB scenario of biological evolution (Fig. 2) is that there is no TOL, even though the tree pattern is a persistent and inevitable feature of life's evolution at numerous stages. The TOL concept is undermined in the most fundamental, physical sense, i.e., under the BBB model, the history of cellular life does not represent a single, interrupted tree of dividing cells [59]. In a close parallel, the evolution of viruses and related selfish elements is not, even in principle, a single tree of replicating genomes [15]. The non-existence of TOL does not mean that trees for individual genes do not exist either. These trees, conceivably, were brought about by the first BBBs that involved the nucleation of RNA and protein structures. However, the BBB model implies two crucial aspects of these trees that are both compatible with empirical data: i) the deep internal branches of gene trees are strongly compressed as dictated by the rapidity of evolution during inflationary phases, and ii) in general, there is no reason for the topologies of the trees for individual genes to be the same in their deep parts, given the rampant reassortment and recombination of genetic elements during the inflationary phases (although, toward the end of an inflationary stage, some gene combinations would become relatively stable, rendering a degree of coherence on some of the gene trees). An implication of these notions is that concatenation of protein sequences in an attempt to enhance the signal and resolve deep phylogenies, a common approach in genome-wide phylogenetic analysis [22,99,100], is a highly suspect practice at best.

The BBB scenario outlined here is linked to, and derived from, directly or indirectly, several previously developed models and concepts in evolutionary biology. Perhaps, the most important one, conceptually, is Woese's idea of a communal stage in the early evolution of life [40,41] which, indeed, corresponds to one of the inflationary phases of evolution postulated here (Table 1). It is remarkable and more than a coincidence that Woese described the emergence of the biological lineages as a phase transition [40]. The model is also compatible, at least, in the broad sense, with Gould's and Eldredge's notion of punctuated equilibrium [5,6]. As concerns the BBB event associated with the origin of eukaryotic supergroup, the present concept is, in general terms, in line with the ideas of Margulis and coworkers on the pivotal role of symbiosis in evolution [80,101,102]. However, the symbioses hypothesized here and leading to the emergence of the eukaryotic supergroups are not to be identified with the series of symbioses that led to the emergence of the eukaryotic cell under the recent hypothesis of Margulis et al [103]; the latter do not seem to find support in available comparative-genomic data.

Like the recent conceptual analysis of Doolittle and Bapteste [59], the BBB model defies the TOL paradigm. However, there is also a notable difference between the two concepts. Essentially, Doolittle and Bapteste submit that the TOL paradigm is invalid inasmuch as there is substantial HGT. This is, indeed, indisputable as long as the TOL is uncompromisingly defined as a tree describing the evolution of entire genomes. However, as suggested previously, the tree representation can be useful even when it reflects the history of a substantial core of genes rather than all genes [22]. It has been shown that such a core of genes that do not appear to undergo frequent HGT exists in major prokaryotic divisions, such as the proteobacteria [104-106]. Accordingly, I propose here that, whereas the tree pattern does not, in principle, apply to the inflationary phase of evolution, trees, understood as the evolutionary scenarios for gene cores, are adequate descriptions of the slower phase.

Recently, Cavalier-Smith developed the notion of "quantum evolution" according to which major cellular innovations in life's history occurred as quantum leaps followed by periods of relative stasis [107]. The central idea of "quantum evolution" might, at first sight, seem very similar to the BBB. However, the fundamental difference is that, according to Cavalier-Smith, quantum evolution occurs solely via rapid accumulation of mutations not via the qualitatively different mechanism of genetic exchange postulated here.

The notion of Big Bang and the term itself have been repeatedly applied in discussions of rapid cladogenesis in biological evolution, in particular, with regard both to the evolution of protein folds [108,109] and to the evolution of various taxa [26,110,111]. I believe, however, that in these studies, Big Bang served more as a general metaphor than a fundamental analogy. Indeed, the traditional Big Bang model does not seem to have any explanatory potential in this context inasmuch as the Big Bang presents The crucial distinction is that the previous invocation of Big Bang dealt with the description of such events in the traditional models of an expanding universe which allowed only a metaphoric juxtaposition of biology and cosmology. The analogy becomes much more precise and informative when the interpretation of the Big Bang in modern inflationary cosmology is invoked instead.

The BBB model, like most concepts at that level of generalization, is hard to test in its entirety but concrete analyses could help both to falsify aspects of the model and to harness additional support. In particular, a definitive resolution of the deep branches in the phylogenetic trees of bacteria, archaea, or eukaryotes would substantially undermine the BBB concept. Conversely, the model suggests that search for additional symbiosis events that could have given rise to the eukaryotic supergroups other than Plantae might have potential, and if successful, would provide a boost to the model.

It remains an open and intriguing question to what extent the BBB model applies to other transitions in biological evolution beyond those discussed above, e.g., the emergence of animal phyla during (or before) the Cambrian explosion, and what would be the mechanisms underpinning the inflationary phase for these transitions. It seems tempting and potentially fruitful to examine such stages of evolution for possible mechanisms of genetic exchange to account for an inflationary phase; invasions of mobile elements (including viruses) could be one such mechanism. Additionally or alternatively, rewiring of the kernels of regulatory networks that is thought to underlie the divergence of animal phyla [112,113] could be a qualitatively distinct evolutionary force triggering a BBB. More generally, understanding the inflationary phases and the exact processes occurring during BBBs emerges as a major goal of evolutionary biology.

## Reviewers' comments

### Reviewer 1: William Martin (University of Duesseldorf)

If these ideas are drawn from cosmology, that could be stated, but I don't think they are. Instead it seems that these ideas emerge independently from cosmological considerations and are founded in evolutionary thinking, not in cosmological.

**Author's response: ***The ideas, in large part, are, indeed, drawn from cosmology – more precisely, from the juxtaposition of specific models of modern cosmology and biological evolution. In the revised text, the cosmological connection is presented firmly and exclusively as an analogy, albeit a deep one. I should emphasize (and this was made more explicit in the revised manuscript) that I draw a fundamental analogy between biological transitions and the interpretation of the Big Bang in modern, inflationary cosmology. The analogy does not work with the traditional notion of the Big Bang. Therefore, without, at least, a general understanding of the distinction between these two dramatically different notions of the Big Bang, it is hard to understand the analogy and the paper as a whole. Hence the discussion of cosmology here required a special section, albeit a brief one*.

Abstract: "It is proposed that each major transition during evolution that shows the "explosive" pattern of emergence of new classes of biological entities corresponds to a boundary between two qualitatively distinct evolutionary processes (phases)" I think that some specification is needed here, because different biologists have different views as to what the major transitions are.

**Author's response: ***I have tried to explain what is meant by transitions but also indicated that it is, indeed, hard to converge on a complete, definitive list of these*.

Abstract: "that the eukaryotic supergroups emerged through distinct endosymbiotic events". What eukaryote phylogeny buffs are now calling supergroups are things like excavates, opisthokonts, archaeplastida, etc. These are summarized in recent reviews, especially the one by Adl et al cited in (44). They all have only one and the same endosymbiont (the mitochondrion), so distinct symbioses is not at all a clear term here. Plants had a second distinct symbiosis, some algae had distinct 2° symbioses, but that's about it, it seems. So the passage needs clarification.

**Author's response: ***Phrasing was less than precise in the original abstract. Clarifications added, both in the abstract and in the main text. Yes, I speculate on additional symbioses, even if they are not discussed by "specialists", and I make it explicit in the revision*.

"identification of symbiotic events that would have led to the emergence of eukaryotic kingdoms" comment as above.

**Author's response: ***See above*.

In the first two paragraphs of "Background" we see that the tree concept is being contrasted to a rate concept (gradualism). That problem occurs throughout the paper. One cannot easily present rates plus mechanisms (Bangs) as alternatives to shapes (the tree). I don't really have a suggestion as to how to fix this problem of the present paper except for major recouching of the issues. But I do think that it needs to be fixed.

**Author's response: ***This is an important point, and I attempted to make it explicit in several places in the revised manuscript. What I mean is not just a major difference in rate but a difference in mechanism. The underlying mechanism in tree phases of evolution is vertical inheritance resulting in cladogenesis. The underlying mechanism in inflationary stages is exchange, recombination etc such that organismal lineages do not exist. The paper is not just about the fallacy of gradualism (something that, indeed, has been emphasized by Gould-Eldredge, Cavalier-Smith and others). The distinction between the two phases of evolution is not one of quantity but one of kind. I agree that this was insufficiently stressed in the original manuscript, and I attempted to rectify this in the revision*.

Abstract: "In each major class of biological objects, the principal types emerge "ready-made", and intermediate grades cannot be identified." Ouch, that will be up on ID websites faster than one can bat an eye.

**Author's response: ***Here I do not really understand the concern. I changed "ready-made" to "abruptly", to avoid any ID allusions and added clarifications but, beyond that, there is little I can do because this is an important sentence that accurately and clearly portrays a crucial and, to the very best of my understanding, real feature of evolutionary transitions. Will this be used by the ID camp? Perhaps – if they read that far into the paper. However, I am afraid that, if our goal as evolutionary biologists is to avoid providing any grist for the ID mill, we should simply claim that Darwin, "in principle", solved all the problems of the origin of biological complexity in his eye story, and only minor details remain to be filled in. Actually, I think the position of some ultra-darwinists is pretty close to that. However, I believe that this is totally counter-productive and such a notion is outright false. And, the ID folks are clever in their own perverse way, they see through such false simplicity and seize on it. I think we (students of evolution) should openly admit that emergence of new levels of complexity is a complex problem and should try to work out solutions some of which could be distinctly non-orthodox; ID, however, does not happen to be a viable solution to any problem. I think this is my approach here and elsewhere*.

Regarding the animal phyla, see two new papers on Canfield oceans, where they say that the lineages just got larger 580 MY ago.

Fike DA, Grotzinger JP, Pratt LM, Summons RE (2006) Oxidation of the Ediacaran ocean. Nature 444:744–747

Canfield DE, Poulton SW, Narbonne GM (2007) Late-Neoproterozoic deep-ocean oxygenation and the rise of animal life. Science 315:92–95

**Author's response: ***These particular papers seem only tangentially relevant but several others that claim early divergence of animal phyla and those that criticize that claim are cited in the revision*.

Background, the last paragraph of the 1^st ^subsection: "Here, I argue for a fundamentally different solution, i.e., that a single, uninterrupted TOL does not exist." I think we are hitting a big snag here that needs a couple of paragraphs of explanation. Ford Doolittle and others are saying that too, but for very different reasons (for example Doolittle and Bapteste, 2007, PNAS), so it is really necessary to explain what the difference is. I really do think that the essence of the present paper is a general assault on gradualism. The people who most vociferously criticize the ToL concept are saying that i) the ToL is not a tree because lateral gene transfer is widespread and possibly the predominating factor in the overall evolution of prokaryotic gene combinations across chromosomes and that endosymbiosis with endosymbiotic gene transfer is important. They are saying that the ToL is not a tree because it is not, in the main, bifurcating. This paper is not commenting on the topology or general shape of the ToL, but is instead using the term "ToL" without further qualification, hence accepting that a ToL exists in nature (see here Doolittle and Baptest 2007 for the difference between really existing ToLs in nature that one might discover and assumedly existing ToLs evidence for which one might seek), and within a ToL framework is saying that there are order of magnitude differences in the yearly rate and nature at which at which growth occurs along the branches. That comes to the fore with allusions to punctuated equilibrium.

**Author's response: ***Actually, I think herein lies the big misunderstanding and/or the big disagreement. I do not see at all how the concept presented in this paper is compatible with the existence of a "single, uninterrupted" TOL. And, I do not see how any other concept of TOL would be worth of that name. In the revised version, I try to clarify this in several places*.

The cosmology part of the paper would need to be toned down because even if the cosmology parallels can be drawn, the substance of the paper needs to stand or fall on biological arguments, cosmology provides no help at all.

**Author's response:***It has been toned down in the revision (see also above)*.

I think that the discussion of the proposed symbiotic events needs to be contrasted and compared to what Lynn Margulis is saying for example in Proc Natl Acad Sci USA. 103:13080–10385 (2006).

**Author's response: ***Contrasted and compared in the Discussion*.

Section on Cosmology and Discussion: I do not agree that any general similarities between biological process and cosmological process can be drawn. If biologists first have to read up on their cosmology to understand evolution, then something must be wrong. Biologists have long listened to chemists and physicists when it comes to early evolution and its principles. I am convinced that biology and biochemistry are sufficiently rich as disciplines to allow the problems to be cast in biological and biochemical terms. That is a plea for a more down-to-Earth presentation of the present text.

**Author's response:***The presentation was made somewhat more down-to-Earth in the revision but only so much because I am still convinced that there is a substantial and informative (as I see it) analogy between transitional stages of biological evolution and evolution of the multiverse. I actually think it would be very, very useful to any evolutionary theorist to familiarize her/himself with modern cosmology (I am tempted to suggest that is, indeed, necessary, but probably, I should not go that far). In the very least, many fruitful ideas come out of juxtaposing the two fields. By the way, cosmologists are well aware of existing parallels and use them often (Lee Smolin with his cosmic selection – regardless of the ultimate validity of his theory, it is certainly respectable – is the best example). Of course, any scientist is entitled to stay away from any such far-reaching analogies but, in all fairness, those who are poised to pursue them, should have the right to do so as well*.

I think that this whole paper needs to be contrasted to previously published literature on LGT, on punctuated evolution, and on endosymbiotic theory in more detail. I think that most readers will come away with the same feeling. For topics as broad as this MS is cut out, anything short of a book is probably too brief. The easy fix is to focus on a specific point like gradualism.

**Author's response: ***The point about the book is well taken, too (I am, actually, working on one). However, I think that anything that requires a book for a full presentation also can be presented as an "extended abstract" (see Darwin, Charles). This paper is just such an abstract. Nevertheless, some extra references and a few extra words on each of the aforementioned issues were added in revision*.

### Reviewer 2: Sergei Maslov (Brookhaven National Laboratory)

The manuscript by Eugene Koonin describes a compelling analogy between several major evolutionary transitions in the history of life (referred to as Biological Big Bangs or BBBs) and the inflationary model describing the early dynamics of our Universe (or rather the multiverse) following the cosmological Big Bang. What conceptually unifies these two rather disparate phenomena is that both start with a chaotic phase in which a large number of new entities is rapidly created. Following a sharp crossover this phase is replaced with a much slower phase in which these entities are being tested and gradually refined. I liked the philosophical message of the manuscript that major innovations usually start with a chaotic, messy, "evolutionary brainstorming" phase in which freshly generated, still imperfect elements are being freely exchanged and recombined.

The present manuscript also nicely extends/compliments the recently published anthropic principle manifesto by the same author (Biology Direct 2, 15 (2007)).

One question I would like to see addressed in more detail is to what extent the BBBs have to involve extensive genetic recombination. In the introduction chapter of this manuscript the Cambrian explosion is listed among the greatest evolutionary transition (which it undoubtedly was). However, this evolutionary transition is later omitted from the list of BBBs discussed in detail in this manuscript. My guess this is because it does not easily fit the scenario of a rampant genetic exchange. More primitive multi-cellular organisms were in existence well before the Cambrian era and it is hard to imagine a scenario in which multi-cellular organisms would be involved in a large-scale swap of genetic material. On the other hand, it is becoming progressively more and more clear that during (or immediately before) the Cambrian explosion Nature experimented with different schemes of wiring the regulatory network for embryonic development (see e.g. the concept of regulatory "kernels" responsible for different animal body plans in E.H. Davidson, and D. H. Erwin, Science 311, 796 (2006)). Thus a more traditional explanation of the Cambrian explosion involves no rampant horizontal gene transfer but just a very rapid burst of innovation and diversification following the appearance of a new scheme for information processing (in this case represented by the regulatory network orchestrating the embryonic development).

I would very much like Eugene to share his thoughts on whether he feels that a large-scale genetic recombination is in his opinion a necessary condition for a Biological Big Bang to occur?

**Author's response**: *Certainly, I do not insist on a large-scale swap of genetic information (i.e, HGT) being the underlying cause of the emergence of the animal phyla. I suggest leaving the causes of the acceleration of evolution in this and other late transitions wide open. I would suspect that bursts of mobile element dissemination could be important as briefly mentioned at the end of the paper, and possibly, other forms of information exchange. The issue of network rewiring is also touched upon in the revision, with two papers of Eric Davidson cited, and in this context, the possibility of causes of BBBs distinct from genetic exchange is acknowledged. However, a truly detailed discussion of animal evolution is outside the scope of this paper*.

If it is not I could imagine extending the analogy even further to describe rapid technological transitions such as (most recently) the appearance of the Internet and the World Wide Web. Here again the singular act of invention of a new information processing tool was followed by a burst of innovation utilizing it in every imaginable way. As in any of the BBB described in the manuscript in this case the initial rapid innovative phase will inevitably be replaced by a much slower phase in which various "body plans" for Internet-based services are tested and gradually refined (or altogether discarded).

**Author's response**: *Very interesting ideas, indeed*...

### Reviewer 3: Leonid Mirny (Massachusetts Institute of Technology)

This is a very provocative and thought-provoking paper: provocative in putting forward a radical hypothesis of early evolution, and thus provoking to think it over and weight its feasibility. The hypothesis sounds reasonable and very plausible to me. Below I sketch some directions of the theoretical research that can further elaborate evolution through BBBs.

#### Organization

1. I found the manuscript to be very well written but somewhat redundant. I think it will benefit from shortening. E.g., problems that stimulated the development of the model (new folds, viruses, bacteria and archaea, eukaryotic cells and phyla) are listed in several sections of the manuscript. This makes the introduction somewhat lengthy. I wish a reader had a shorter road to the exciting core of the paper (pages 13–16, in my opinion).

**Author's response**: *I understand the sentiment but these are highly non-orthodox ideas, so I believe the long introduction should be helpful for most readers*.

#### Questions/suggestions

1. The emergence of the new folds in attributed to the first BBB. I would think that each BBB leads to the emergence of new genes and folds, specifically the one that lead to the emergence of the eukaryotic cell with its unique eukaryotic genes and folds.

**Author's response**: *There seems to be a bit of a misunderstanding here. Certainly, there was some (though, limited) emergence of new folds associated with different BBBs. However, the first BBB involved the emergence of the very first distinct protein (and RNA) folds*.

2. The emergence of eukaryotic cell and eukaryotic lineages are put together into a single BBB. The emergence of the bacteria and formation of bacteria lineages, in contrast, are split into two distinct BBBs. Perhaps eukaryotic BBB can be thought of as a two-stage process: (i) the emergence of the proto-eukaryotic cell through multiple symbiotic events and expansion of its population, followed by (ii) the formation of the major eukaryotic lineages through continued swallowing of other cells. The proto-eukaryotic cell may contain the hallmark of the eukaryotes (e.g. nucleus, chromatin etc), while missing some specific organelles that distinct the lineages (e.g. chloroplasts). An alternative view would be that both processes were happening independently and at the same time. This possibility sounds questionable, as it may not be able to explain the common features of all eukaryotic cells. I think description of this BBB needs some clarification.

**Author's response**: *Yes, I envisage this BBB more like a two-stage process. A variety of amendments and clarifications were made to the description, in part, also, in response to the criticisms of Reviewer #1*.

3. Emergence of the protein synthesis – one of the most puzzling, to my mind, event in evolution, remains untouched in this new picture of evolution leaving it to be invented in the bubbling guts of the hydrothermal vents. I wonder whether the BBB view of evolution can shed light on this mystery.

**Author's response**: *Of course, the origin of protein synthesis (a great challenge, indeed!) is related to the first BBB. Beyond that, however, I am not immediatel sure how the BBB model could help. For two complementary perspectives, see my recent papers*: Wolf, Y. I., Koonin, E. V. On the origin of the translation system and the genetic code in the RNA world by means of natural selection, exaptation, and subfunctionalization. *Biology Direct *2007, **2:**14, and Koonin, E. V. The cosmological model of eternal inflation and the transition from chance to biological evolution in the history of life. *Biology Direct *2007, **2:**15, and

#### Some thoughts

##### 1. Evolution with extensive HGT

An intriguing theoretical problem is to develop a mathematical model of evolution in the population of cells through extensive HGT (or, broadly, with "leaky" cell walls and rapid exchange of genetic material). Intuitively, evolutionary processes in such communities can be quite different from the picture drawn by the classical population genetics. Consider dynamics of an advantageous new gene in such (HGT+ leaky cells) population. The spread of this gene will be driven by two processes: growth of the sub-population of cells carrying this gene (classical picture) and faster and faster spread of the gene through HGT as the sub-population carrying the gene growth. Taken together these processes can lead to more rapid (than in the classical picture) fixation of an advantageous allele. Thus it is not only the mere fact of extensive production of the new genetic material through HGT, but also the underlying dynamics that can make BBBs effective melting pots of evolution.

I would also expect such process (leaky cells+HGT) to be more efficient than evolution in compartmentalized cell-free communities of molecules in hydrothermal vents (HGT only).

**Author's response**: *Yes, I quite agree, hopefully, we will be able to develop a mathematical model in a reasonable future*.

##### 2. Mechanism and causes of "heating" and "cooling"

The next piece of the puzzle to be sought is the understanding of the mechanisms that lead to initiation and termination of BBBs. To my mind, the key question is whether periods of BBB have been triggered by some environmental events or by spontaneous nucleation (or both).

**Author's response**: *Yes, I think both factors were important*.

One possibility is that a population model of BBBs (leaky cells+HGT) may lead to a possible solution of the problem by suggesting mechanisms of spontaneous initiation and termination of BBBs. For example, the winners in the BBB evolution (i.e. cells that reached some local maximum of fitness) may benefit from sealing their cell walls and thus leaving the community to (i) prevent further recombination events that may ruin their local advantage; (ii) stop sharing of the advantageous genomes with other cells to secure their own advantage. It would be interesting to see whether a model of BBB where cells may stochastically switch from an altruistic (open walls) to a selfish (closed walls) behavior lead to spontaneous "sporulation" of selfish wall-sealed cells that have reached locally optimal fitness, thus leading to effective "cooling" of a BBB.

**Author's response**: *Again, I agree, it will be interesting and, hopefully, feasible to develop such models*.

As for the initiation of BBB, the author proposes that the small population size may be lead to inventions due to fixation of otherwise deleterious mutations. Perhaps, more generally, one can think of numerous populations that undergo Muller ratchet catastrophes, thus shrinking and leading to this "last whisper" inventiveness. One of which may end up inventing a mechanism for BBB (e.g. swallowing other cells).

## Authors' contributions

EVK developed the model and wrote the manuscript.
